# Occurrence of Malformations of the Upper Extremity in Tibial Hemimelia: Correlation with the Jones Classification

**DOI:** 10.1007/s43465-025-01359-9

**Published:** 2025-03-21

**Authors:** Lisa-Marie Seeor, Albert Fujak, Chakravarthy U. Dussa

**Affiliations:** 1 Department of Trauma and Orthopaedic Surgery, Pediatric and Neuro Orthopaedics, University Hospital Erlangen, Friedrich-Alexander-Universität Erlangen-Nürnberg, Krankenhausstr. 12, 91054 Erlangen, Bavaria Germany; 2 Paediatric and Neuro-Orthopaedics, Department of Orthopaedics and Traumatology, Ludwig-Maximillian-Universität, Klinikum Großhadern, Marchioninistrasse 15, 81377 Munich, Bavaria Germany

**Keywords:** Tibial hemimelia, Tibial aplasia, Jones classification, Upper limb, Syndrome, Limb malformation, Classification

## Abstract

**Background:**

Tibial hemimelia is a rare malformation with a wide clinical spectrum of presentation. The severity of this condition can be typed using different classification systems. It can exist as an independent entity or can be associated with upper limb or visceral malformations. The aims of our study are therefore, a. to report the incidence of upper limb deformities in relation to the severity of tibial hemimelia classified by the Jones classification, b. incidence of tibial hemimelia as a part of a syndrome c. to report the overall incidence of the associated upper limb and visceral deformities.

**Methods:**

A retrospective study was done using radiographs and clinical notes. The severity of the tibia deformity was assessed using the Jones classification. The clinical notes were reviewed to report the additional findings in the upper limbs and the visceral organs.

**Results:**

The study included 69 patients with tibial hemimelia aged from 10 months to 34 years. Twenty of them (28.9%) had bilateral involvement. Additional malformations were observed in 56 patients (81%) involving the upper and lower limb and visceral organs. In 11 patients (16%), tibial hemimelia occurred as part of a syndrome, most often being Gollop–Wolfgang complex. The incidence of malformations of the upper extremities was 15 (21.7%), four of which (26.6%) involved bilateral upper extremity malformation. The cleft hand was the most frequent malformation of the upper extremities, followed by hypoplasia or aplasia of the thumb and fingers.

**Conclusion:**

Jones type I tibial hemimelia is often associated with visceral and upper limb malformations. visceral anomalies are associated with syndromal forms of Tibiail hemimelia. Several forms of upper limb malformations with varying severity were associated with the disorder. Therefore, a holistic approach to the patient should be initiated soon after birth involving a paediatric, hand and visceral surgeon, to provide the best possible care.

**Level of evidence:**

Level IV study, retrospective review of 69 patients with tibial hemimelia.

## Introduction

Tibial hemimelia is a relatively rare congenital malformation with a prevalence of 0.21 per 10,000 births [1]. Billroth was the first to correctly describe the condition in 1861, which was later revised by Dankmeijer [2, 3]. The aetiology of tibial hemimelia nevertheless continues to be uncertain, although autosomal-dominant and recessive inheritance, has been suggested [4, 5]. Tibial hemimelia can manifest as an isolated malformation or as part of a syndrome. A syndrome is defined as a medical condition that is characterised by a particular complex of symptoms. It is diagnosed by physical examination after birth supported by an X-ray. The clinical spectrum varies from a complete absence of the tibia to its underdevelopment to varying extents, and unilateral as well as bilateral cases are possible. About 60% of the patients have associated congenital malformations of the lower limb, visceral organs and upper limbs [6]. Several syndromes have been associated with tibial hemimelia such as Werner syndrome [7], Langer–Giedion syndrome [8], Gollop–Wolfgang complex [9], CHARGE syndrome [10] or VACTERL syndrome [11].

Several classification systems are available to describe the wide manifestations of tibial hemimelia depending on the severity. The first attempt to classify tibial hemimelia was made by Jones in 1978 [12], which was modified by Kalamchi and Dawe in 1985 [13]. The drawback of both the above classifications is, their inability to accommodate all the phenotypic manifestations of tibial hemimelia. Keeping the deficiencies of both the classifications in view, Weber in 2008 put forward a more extensive classification and scoring system which takes the deformities of the entire limb into consideration [14], but it is cumbersome to use in day-to-day practise. Paley described yet another classification in 2003 [15] and modified in 2015 [16] and rerevised in 2016, [17] although being equally cumbersome, has been said to have therapeutic relevance. Depending on the severity of the condition, a wide variety of therapeutic options ranging from conservative methods such as prosthetic fitting to surgical reconstruction is available [18, 19].

Although the malformations of the upper extremities have been described in association with tibial hemimelia, they have been not extensively studied until now [17, 31]. Even the incidence of visceral malformations with tibial hemimelia are not been reported. Keeping this in view, it is worthwhile determining, if the occurrence of the malformations in the upper limbs have a relation to the severity of expression of tibial hemimelia. Therefore, the aims of this study were a. to report the incidence of upper limb deformities in relation to the severity of tibial hemimelia classified by Jones classification, b. incidence of tibial hemimelia as a part of syndrome and c. to report the overall incidence of the associated upper limb and visceral deformities.

## Material and Methods

A retrospective study was done at a single centre. All cases of tibial hemimelia treated at this centre between 2006 and 2021 were included in the study. The study was approved by the Clinical Ethics Committee of the Faculty of Medicine at Friedrich-Alexander Universität Erlangen-Nürnberg in Germany (21-462-Br).

The exclusion criteria were absence of X-rays or CT scans, lack of documentation on the lower limb, upper limb and visceral malformations. Those tibial hemimelias, which could not be classified according to Jones, were placed in a separate group called ‘unclassified’.

For this purpose, clinical records, and available radiographs were used. Anterio-posterior and lateral radiographs of the lower limb were used to classify the type of tibial hemimelia. Where CT scans were available, they were used. Jones classification was used to classify the tibial hemimelia, as this is easy, and the classification is repeatable (Table 1) [12]. All cases could be evaluated according to Jones classification and satisfactorily allocated to one of the five groups (including unclassified group) The children without appropriate radiographs or lack of CT scans or Children who were lost for follow-up were excluded from the study. In this paper we used the following definition for a syndrome; “A syndrome is a medical condition that is characterised by a particular group of signs and symptoms” (Collins dictionary). In fact, the diagnosis of a syndrome was already established at the referring hospital, before presenting to our hospital. No efforts were made to trace the genetic diagnosis of these children and therefore, was not the basis for the diagnosis of a syndrome. The phenotypic presentation of multiple malformations (phenotype) in a child were cross-checked before accepting the established diagnosis of a syndrome. The visceral anomalies were recorded from the notes of the referring hospital. The upper limb and lower limb deformities were both clinically and radiologically examined. Patients records were not looked for the treatment, results of the treatment and function of the upper and lower limb, as this was not the purpose of the study.

**Table 1:** Characteristics of each type of tibial hemimelia according to the Jones classification [16–18]

Type	Characteristic(s)
I	Total absence of the tibia
II	Presence of proximal tibia, fibula dislocated proximally
III	Presence of distal tibia, hypoplastic proximal tibial epiphysis
IV	Shortened tibia with tibiofibular diastasis, dislocation of proximal fibula, overlength of fibula

## Results

Between 2006 and 2021, 92 patients were treated for tibial hemimelia at the reference paediatric orthopaedic hospital. As per the exclusion criteria, 23 subjects were excluded. Therefore, 69 subjects were included in the study. Of these, 45 were males and 24 were female. Their age ranged from 10 months to 34 years.

**Fig. 1:**
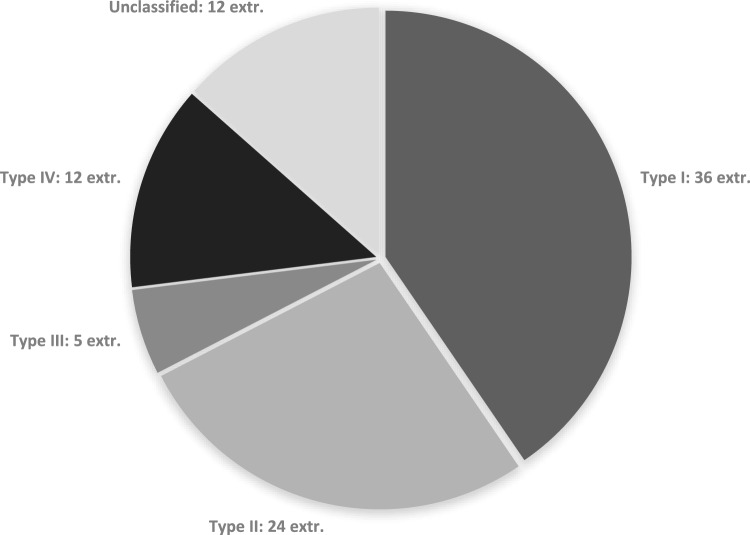
Division of tibial hemimelia in 89 extremities of 69 patients according to the Jones classification. *Note.* *extr.*  extremities

Unilateral involvement was the common presentation and was seen in 49 subjects (71%). A bilateral involvement was seen in 20 subjects (29%). Thus, a total of 89 extremities with tibial hemimelia were analyzed in 69 subjects. In Unilateral cases, right side (59%) was more affected than the left (41%). The distribution of limbs according to Jones classification is presented in Fig. 1. Congenital malformations of the limbs and visceral organs were seen in 56 subjects, constituting 81% of the study group subjects. The congenital anomalies of the extremities were more than those for the visceral organs. Forty subjects (62%) showed anomalies of musculoskeletal system, and three (4%) showed visceral anomalies alone. Both musculoskeletal and visceral malformations were observed in ten subjects (14%). A list of all additional musculoskeletal and visceral malformations, except the ones on upper limbs, are presented in Table 2.

**Table 2:** Types of all musculoskeletal and visceral malformations and presentation of all additional malformations in our patients with tibial hemimelia

Musculoskeletal malformations	*n*
Lower limb (proximal to distal)	
Proximal femoral focal deficiency on the contralateral side (congenital short femur/ PFFD)	7
Hypoplastic fibula (these were seen in association with congenital short femur/ PFFD on the contralateral side)	6
Foot with 1 ray	2
Foot with 2 rays	2
Foot with 3 rays	2
Foot with 4 rays	9
Foot with 6 rays	6
Foot with 7 rays	1
Clubfoot	10
Cleft foot	3
Splayfoot	1
Pes calcaneus	1
Pes equinus	8
Hallux valgus	2
Other	
Hip dysplasia	7
Scoliosis	7
Tethered cord syndrome	2
Torticollis	1

Visceral malformations	*n*
Hypoplastic right heart syndrome with pulmonary and tricuspid valve atresia	1
Fallot’s tetralogy	2
Dextrocardia	1
Anal atresia	3
Hypoplastic colon	1
Kidney agenesia	5
Ectopic pelvic kidney	1

The tibial hemimelia was a part of syndrome in 11 subjects (16%), of which Gollop–Wolfgang complex, was most common. It was present in six subjects (9%). This was followed by VACTERL syndrome in four subjects (6%) and Werner syndrome was seen in one subject.

Upper limb anomalies were seen in 15 subjects (22%) are presented in Table 3. Cleft hand was the most common malformation of the upper limbs, followed by hypoplastic or aplastic fingers or rays. Other upper limb malformations observed were radial hexadactyly, syndactyly, radius aplasia, elbow flexion contracture, clinodactyly, and supination contracture of the forearm. The recommended surgical procedures for the treatment of upper limb anomalies are are presented in Table 4.

**Table 3:** Presentation of all patients with Jones classification of tibial hemimelia and additional upper limb malformations

Pat	Sex	Side Tib Hem	Jones	Upper limb anomalies	Side of upper limb
**1**	M	L	II	Radial hexadactyly	R
**2**	M	L	III	Cleft hand	L
**3**	M	R	Uncl	Syndactyly DII and III	L
		L	II		
**4**	M	R	III	Hypoplastic DII, aplastic DIII and IV	L
		L	III		
**5**	M	L	I	Radius aplasia, aplastic DI and II	L
**6**	M	L	II	Elbow flexion contracture	B
**7**	F	R	II	1 ray hand and elbow flexion contracture	R
				Cleft hand	L
**8**	M	R	Uncl	Cleft hand, radius aplasia	R
		L	I	Cleft hand, radius aplasia	L
**9**	F	R	Uncl	Cleft hand	R
		L	I	Cleft hand	L
**10**	F	R	II	Radial hexadactyly	R
		L	IV		
**11**	M	R	Uncl	Cleft hand	R
		L	I		
**12**	F	R	I	Hypoplastic DI	R
**13**	M	R	Uncl	Clinodactyly DV	R
**14**	M	R	I	Cleft hand, radius aplasia, aplasia DI	R
**15**	M	L	I	Supination contracture	L

*Note.* *Pat.* Patient, *M* male; *F* female; *R* right; *L* left; *B* Both sides, Side Tib. Hem. = Side of tibial hemimelia, Uncl. = Unclassified, Side of Upp. Li = side of upper limb anomaly

**Table 4:** Summary of recommended surgical procedures for the upper limb malformations found in our patients 20–30

Upper limb malformation	Pre-operative considerations	Surgical procedures	Age recommended for surgery
Cleft hand	- Partial cleft or complete cleft with functional limitation - Aesthetic aspects	- Z-plasty for partial cleft hand	3–5 years
		- Snow-Littler reconstruction of the thumb web space with volar rotational flaps and transposition of the index ray	3–5 years
		- Dorsal rotational flaps for major deficiencies	2–5 years
Hypoplastic/ aplastic thumb	- Mild hypoplasia with stable carpometacarpal joint (CMCJ) - Severe hypoplasia with instable CMCJ - Aplalsia	- None	
		- Stabilization of CMCJ	2–4 years
		- Pollisisation and Opponensplasty	2–4 years
		- Non-vascularized bone grafts	2–4 years
		- Microsurgical toe-to-thumb transfers	2–4 years
Radial hexadactyly	- Rudimentary thumb - Complete or partial duplication	- Excision or ligation - Excision of underdeveloped thumb and soft-tissue reconstruction	At the age of 1 year At the age of 1 year
Syndactyly	- Partial - Complete	- Digital separation and full-thickness skin grafts - Graftless techniques with dorsal metacarpal artery flap (Sherif technique)	At the age of 1 year At the age of 1 year
Radial longitudinal deficiency/ radius aplasia	- Intervention depends on the extent of malformation and the function	- Buck-Gramcko procedure with transfer of the radial wrist muscles on the ulnar wrist	6 months–5 years
Elbow flexion contracture	- > 30° with functional limitations - Ankylosis	- Open ventral arthrotomy ± soft-tissue lengthening - No surgery	6 months–5 years
Clinodactyly	- No pain or functional limitation - Severe angulation with functional limitation	- Conservative	
		- Physiolysis	2–5 years
		- Osteotomies	6–10 years or earlier
Supination contracture	- Functional limitation	- Soft tissue or bony procedures	2–5 years

The involvement of upper limbs was unilateral in 11 subjects and 4 subjects, the involvement was bilateral. Of the four subjects with bilateral involvement of upper limb, two subjects had a bilateral tibial hemimelia and in other two subjects, a unilateral tibial hemimelia was seen. On contrary, a bilateral tibial hemimelia was seen in six subjects with a unilateral upper limb malformation, and the rest nine of the subjects a unilateral tibial hemimelia was seen. In relation to the tibial hemimelia, the malformation of the upper limb occurred on the ipsilateral side in 6, and in 2 subjects a bilateral malformation.

In 13/15 subjects with upper limb malformation, the malformation was isolated. However, a combination of malformations of upper limb were seen in 2/15 subjects. These included an ipsilateral radius agenesis, a cleft hand and aplasia of the first digit in one subject and a left-sided cleft hand and a right-sided single-ray hand and an elbow flexion contracture.

Of the 15 subjects with upper limb malformations, two were diagnosed with a syndrome: one with VACTERL syndrome and the other with both VACTERL syndrome and Gollop–Wolfgang complex. The other 13 subjects showed additional musculoskeletal malformations of lower limbs and spine. The most frequent were clubfoot and 4-, 3-, or 2-ray foot, followed by scoliosis and hip dysplasia. The frequency of the additional deformities has been depicted in Table 2.

**Fig. 2:**
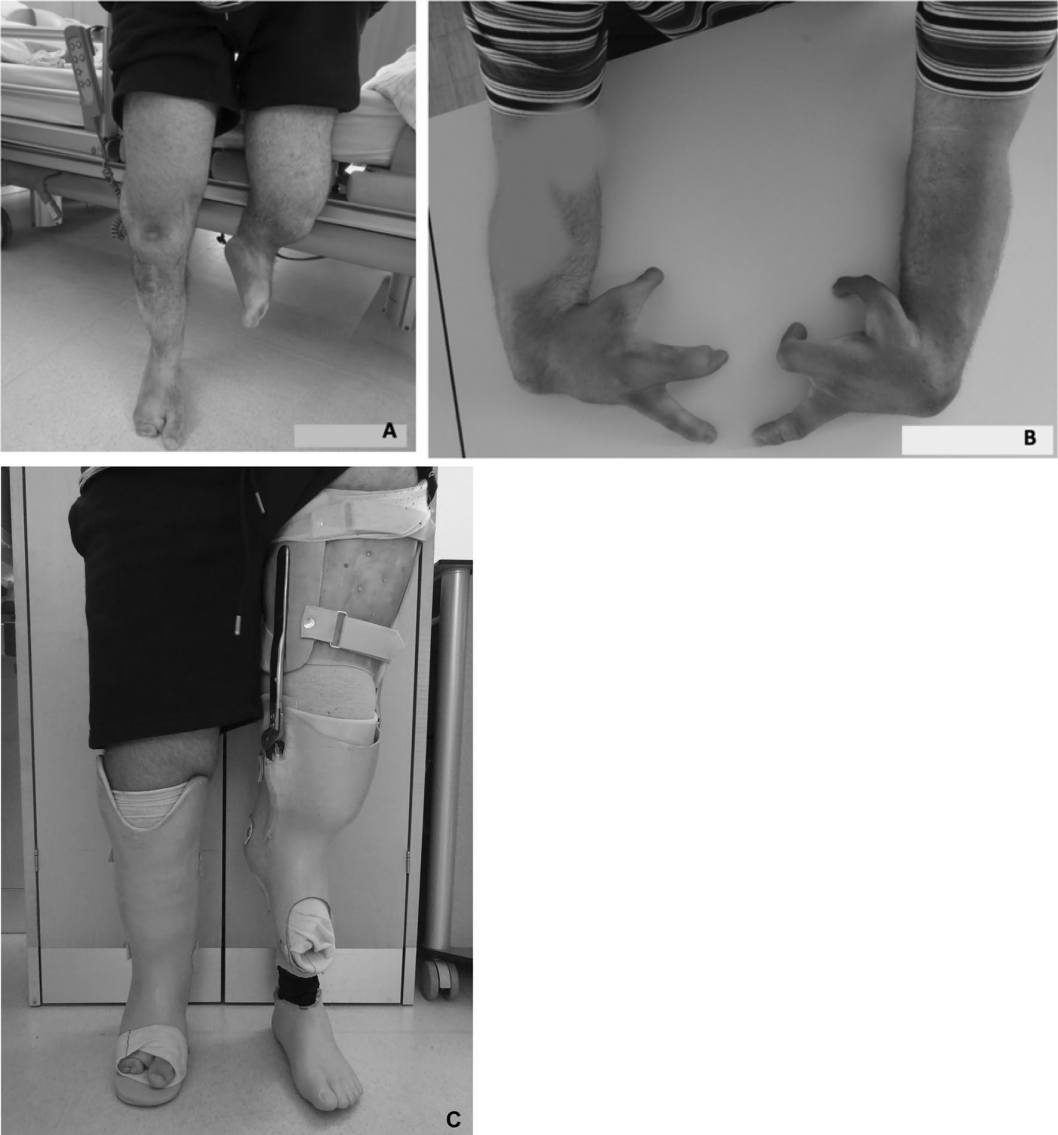
Clinical photographs of a 21-year old man with tibial hemimelia and malformations of the upper limb: **A** tibial hemimelia type II according to the Jones classification on the left side and type IV on the right side, without any surgical correction made; **B** cleft hands and radial aplasia on both hands, which have been operated on several times; and **C** orthotic fitting of the right leg and prosthetic fitting of the left leg with extension above the knee of the patient’s lower extremity

The 15 subjects in this study sample with upper extremity malformations had a total of 21 lower extremities affected by tibial hemimelia. Correlating the upper limb deformities to Jones classification, the upper limb deformities occurred most with Jones type I tibial hemimelia, followed by type II and unclassified type. The number of upper limb deformities in relation to Jones classification are presented in Table 5. The clinical findings of one subject with bilateral involvement with upper and lower limbs is depicted in Fig. 2.

**Table 5:** Incidence of upper limb deformities according to Jones classification

Tibial hemimelia according to Jones	Upper limb malformations Yes	Upper limb malformations No	Total number of limbs *N* = 89
I	7	29	36
II	5	19	24
III	3	2	5
IV	1	11	12
Unclassified	5	7	12
	21	68	89

## Discussion

The study showed a 21.7% incidence of upper limb anomalies in subjects with tibial hemimelia and mostly associated with Jones type I tibial hemimelia. In 8% of the subjects, the tibial hemimelia was associated with a syndrome with cardiac and gastrointestinal malformations being common. In all 81% subjects with tibial hemimelia showed associated malformations.

The distribution of types of tibial hemimelia in this study with 69 subjects concur with the results of the Jones study. Similar to the Jones study, we found type I with an incidence of 40%, to be the most frequent type of tibial hemimelia [12]. Analysing 20 patients with 29 affected extremities, Jones also found type I to be most frequent with 62%, followed by type II with 17% and type IV with 14%. The rarest type according to Jones was type III with 7% [12]. With 6% in the present study, the incidence of Type III was similar to that reported by Jones and Weber [12, 14]. On contrary Clinton and Birch could not assign any of the limbs in their study with 125 affected extremities in 95 patients to Type III [31]. They found 63% of the extremities could be assigned to type I, 14% to type II and 10% to type IV tibial hemimelia [31]. Weber studying 63 patients with 95 affected assigned extremities 61% to type I, 16% to type II, 3% to type III and 5% to type IV [14]. The reason for the difference in distribution of the types of hemimelia is more likely be due to the small sample size.

Despite meticulous examination, 13.4% of the limbs with tibial hemimelia in this study could not be assigned to any of the types in the Jones classification. Similar experiences were made by the authors in other studies [14, 17, 31]. Remarkably, all patients in our study sample who could not be assigned to any type of tibial hemimelia according to Jones classification, had a similar radiological picture. Both proximal and distal tibial epiphyses were present, but the tibia was very much shortened whilst the fibula was relatively overlong. A similar observation was made by Clinton and Birch and concluded that the observed type in his study was similar to that Weber described in types I and V: Type I has been described as hypoplasia of the whole tibia and type V as a biterminal aplasia. [14, 31]

Despite this drawback, Jones classification is easy to use and has therapeutic significance. To overcome this problem, others have developed other more elaborate classification systems, which may be of academic importance but less easily clinically applicable. [14, 17, 31]

Weber developed a new classification addressing this and other presentations of limbs not described by Jones using magnetic resonance tomography (MRI) [14]. Although we did not use a MRI in this study, considering the proportion of shortening of the tibia in relation to fibula, of 12 unclassifiable limbs in this study, 10 limbs showed a phenotype similar to Weber type I, 1 limb similar to Weber type V, and 1 limb similar to Weber type VI.

Several additional malformations of the upper and lower limb and visceral organs were associated with tibial hemimelia. An incidence of 81% additional malformations associated with tibial hemimelia in this study is considerably higher than in the literature [17, 31]. Schoenecker et al. reported 60% of their study group and Clinton and Birch reported 79% of their study group had additional malformations in association with tibial hemimelia. [17, 31]

Gollop–Wolfgang complex, followed by VACTERL syndrome are commonly associated with tibial hemimelia [7, 9, 32]. When the tibial hemimelia is associated with a syndrome, then the clinical presentation is often more complex. Gastrointestinal and cardiac involvement are common when tibial hemimelia is associated with a syndrome and was present in ten subjects with tibial hemimelia constituting 14% of the study population, of which four subjects (6%) belonged to syndrome. The gastrointestinal involvement presents as esophageal and/ or anal atresia. Although several other syndromes were described in combination with tibial hemimelia as mentioned in the introductory section of the paper, none of these syndromes were seen in our study population.

The main focus of this study is to examine the incidence and types of anomalies of upper limb in association with tibial hemimelia. Previous studies which reported upper limb malformations in tibial hemimelia did not study the incidence of upper limb in relation to the Jones classification, as well as the occurrence of upper limb deformities in the syndromic form of tibial hemimelia. The anomalies of upper limb in this study with 21.7% of the subjects was the least in comparison with the literature, with cleft hand being the most common upper limb malformation. In Kalamchi and Dawe’s study with 21 patients, found 29% of their patients had a concurrent upper limb malformation and tibial hemimelia [13]. The prevalence was therefore slightly higher than the present study. Three of these 6 patients had a cleft hand and the other 3 had different anomalies of the thumb, including a bifid thumb, syndactyly, or an absent thumb [13]. Of the 95 subjects in the study from Clinton and Birch, 32 (34%) subjects had upper extremity malformations, the most common one was a cleft hand (15 subjects), followed radial deficiency in eight subjects. Absent fingers were seen in three of their study population, two had an ulnar deficiency, and there was each one case of thumb hypoplasia, transradial deficiency, absent of a hand and elbow pterygium with single-ray hand [31]. Schoenecker et al. analysed 57 subjects with tibial hemimelia of whom 16 (28%) had upper extremity malformations; five subjects with syndactyly and five cleft hand, three with absent or hypoplastic fingers, two with triphalangism of both thumbs and one with radial dysplasia [17]. The occurrence cleft hand showed no frequent occurrence with syndromes and was seen in only one patient with VACTERL syndrome. In contrast, the cleft hand occurred in this study more frequently in bilateral cases of tibial hemimelia. This observation could not be found in other studies.

We also observed a particular pattern in three patients: the presence of bilateral tibial hemimelia with a bilateral cleft hand. In all three cases, tibial hemimelia was classified as Jones type I on one side but could not be classified on the other. The identicalness of constellation of clinical malformations in all the three cases may suggest an identical genetic anomaly/ mutation for the deformity and may represent an unknown syndrome.

Several other malformations of the upper limb reported in this study were already mentioned in the literature [13, 17, 31, 33, 34]. Clinton and Birch, as well as Fernandez-Palazzi et al. mentioned the occurrence of upper limb deformities in association with Jones type I tibial hemimelia [31, 33]. The findings in this present study are in line with the results of both the above mentioned studies: We also could observe additional upper extremity malformation to occur most frequently in patients with Jones type I tibial hemimelia. Therefore, it could be assumed, that a complete absence of tibia is more likely to result in a rather severe form of developmental defect of the upper limb too.

Considering the high incidence of visceral and upper limb malformations, a thought may be given to group the tibial hemimelia in addition to the classification systems, taking the other malformations into consideration. The senior author (CUD) found 3 patterns of tibial hemimelia presentation; a. isolated tibial hemimelia, b. tibial hemimelia with upper limb ± lower limb anomalies and finally, c. tibial hemimelia with syndromes ± upper ± lower limb anomalies.

The main weaknesses of this study are its retrospective nature and lack of MRI as basis for exact classification of this deformity. However, this may have a little clinical relevance. The observer bias in classifying the tibial hemimelia, could occur irrespective of the classification used.

Although the study population is small, considering its rarity of the disease, the study population is comparable to that in the literature. It is possible that the existing literature and the results of this study do not reflect the true incidence of the upper limb deformities in association with tibial hemimelia. Therefore, larger number have to be recruited that is possible only through multi-centre studies. Until now, none of the studies have studied the associated malformations in such a detail. Jones classification has been used to correlate the incidence of upper limb and visceral anomalies. Their incidence would understandably change when other classification systems are used. Since 21.7% of the children had an upper limb malformation of which, 68% of them having a severe malformation of the upper limb, it would be interesting to study the use and function of the upper limb in these patients in the future.

In conclusion, there is a high incidence of upper limb anomalies with varying severity and clinical presentations associated with tibial hemimelia. Cleft hand is the common anomaly. Its bilateral occurrence together with bilateral tibial hemimelia is a interesting presentation and may suggest an underlying unknown syndrome. Involvement of the upper limb bring more challenges to the treating physician, parents and finally the child. A comprehensive evaluation of the patient, including associated malformations and any syndromic conditions, is essential for selecting the optimal treatment and achieving the best functional outcome.
